# Intravascular large B-cell lymphoma presenting with SIAD and pituitary insufficiency: a unifying diagnosis for multiple endocrinopathies

**DOI:** 10.1530/EDM-24-0069

**Published:** 2025-10-31

**Authors:** Zoe Gavey, Raymond Dharmaputra, Nirjhar Nandi, Ashim Sinha

**Affiliations:** ^1^ Department of Endocrinology, Cairns Hospital, Cairns, Queensland, Australia; ^2^ Monash Health, Clayton, Victoria, Australia; ^3^ James Cook University, Cairns, Queensland, Australia

**Keywords:** pituitary, rare diseases/syndromes, neuroendocrinology, SIADH

## Abstract

**Summary:**

Intravascular large B-cell lymphoma is an uncommon haematological condition that may present with a variety of non-specific symptoms and signs. In this report, we discuss the case of a man in his 70s who presented with subacute cognitive and functional decline. Subsequent investigation revealed hyponatraemia secondary to syndrome of inappropriate antidiuresis (SIAD), as well as hypopituitarism. The underlying aetiology for his condition was not discovered until postmortem examination following his death from respiratory failure, which demonstrated intravascular lymphoma involving multiple organs, including the brain, dura and pituitary. As such, this case represents the challenging diagnosis of a rare cause of multiple endocrinopathies.

**Learning points:**

- Intravascular lymphoma is a rare diagnosis, often presenting with non-specific symptoms and biochemical derangements.
- This condition should be considered as a differential diagnosis for hypopituitarism, presenting in isolation or in combination with the syndrome of inappropriate antidiuresis.
- A broad and multisystem differential diagnosis should be considered for any patient presenting with endocrinopathy.

## Background

Intravascular lymphoma is a rare clinical entity, which can be challenging to diagnose given its varied presentation. It is characterised by the growth of lymphoma cells in blood vessels, which can lead to vessel occlusion and organ impairment. Pituitary involvement in this condition is uncommon; however several reported cases have described subsequent hypopituitarism requiring hormone replacement (1). Intravascular lymphoma presenting with SIAD is a rare but reported association, with at least nine cases described in the literature (2). Here we present a man in his 70s who presented initially with hyponatraemia, with investigations suggestive of SIAD but with an unclear aetiology. His subsequent pituitary panel revealed hypopituitarism. Further investigations, as well as his initial presenting symptoms, were suggestive of malignancy, but an obvious primary or recognisable syndrome was not identified to direct treatment. The diagnostic challenge of this case was seeking a unifying diagnosis for his varied presentation, in particular given his multiple endocrine abnormalities.

## Case presentation

A man in his 70s presented with a history of subacute functional decline over 2 months, with weight loss, poor balance, headache, and confusion. His only medical history of note was a recent total knee replacement 5 months prior. He took no regular medications, did not consume any alcohol, and was a non-smoker.

Before his hospital admission, investigations performed by his general practitioner demonstrated mild hyponatraemia (132 mmol/L), with paired urine osmolality of 372 mmol/kg and urine sodium excretion of 72 mmol/L, suggestive of syndrome of inappropriate antidiuresis (SIAD). Other findings included mild anaemia (110 g/L) and thrombocytopenia (150 × 10^9^/L), raised serum ferritin (1,050 μg/L), and an elevated LDH (up to 970 U/L).

The constellation of clinical findings, including weight loss, anaemia, thrombocytopenia, and raised LDH, gave an impression of SIAD from an occult haematological malignancy. Peripheral flow cytometry was unremarkable. Serum protein electrophoresis, urinary Bence-Jones protein, and free light chain analysis were also all negative. Contrast CT of the chest demonstrated a 9 mm nodule on the left costophrenic sulcus and three lytic lesions in the cervical vertebral bodies. A CT of the abdomen and pelvis further demonstrated a 23 mm liver cyst and a moderately enlarged prostate, but no masses were reported. Subsequently, MRI brain was performed, which was again reported as normal.

Following the diagnosis of SIAD, he was commenced on treatment with fluid restriction and salt tablets. In addition, he was prescribed fludrocortisone for management of significant postural hypotension. Unfortunately, his symptoms continued to progress, which led to his hospital presentation due to severe hyponatraemia with confusion (serum sodium of 117 mmol/L).

## Investigation

Table 1 summarises all clinical investigations performed over the course of the patient’s illness.

**Table 1:** Summary of investigations performed.

Test	Result	Reference range
Biochemical panel		
Serum sodium, mmol/L	121	135–145
Serum osmolality, mmol/L	267	275–295
Urine osmolality, mmol/L	372	
Endocrine investigations		
TSH, mIU/L	1.5	0.4–5
Free T4, pmol/L	8.8	10–25
ACTH, ng/L	18	9–51
Short synacthen test (cortisol), nmol/L		
Baseline	590	
30 min	749	
60 min	901	
Growth hormone, µg/L	1.6	
IGF-1, nmol/L	15	7–29
Prolactin, mIU/L	380	<500
LH, U/L	2	<9
FSH, U/L	3	<10
Testosterone, nmol/L	10.1	11.0–40.0
Haematological investigations		
Haemoglobin, g/L	110	120–180
Red cell count, ×10^12^/L	3.61	3.5–6
White cell count, ×10^9^/L	11.5	3.5–11
Neutrophil, ×10^9^/L	9.65	2–8
Lymphocyte, ×10^9^/L	0.45	1–4
Monocyte, ×10^9^/L	1	0.1–1
Basophil, ×10^9^/L	0	<0.2
Eosinophil, ×10^9^/L	0.11	<0.4
Reticulocytes, ×10^9^/L	205	10–100
Myelocytes, ×10^9^/L	0.22	<0.01
Platelet count, ×10^9^/L	150	140–400
LDH, U/L	970	120–250
Iron studies		
Transferrin saturation, %	17	
Ferritin, µg/L	1050	
Peripheral blood flow cytometry		No evidence of haematological malignancy
Lymphocytes	11%	
T cells	90%	
CD4/8 ratio	5.7	
B cells	4%	
k/L ratio	1	
NK cells	6%	
Whole body CT-PET scan	No FDG avid esion detected	
Immunological investigations		
Anti-VGKC	Negative	
Anti-GAD	Negative	
Anti-NMDA	Negative	
Anti-Hu	Negative	
Anti-Ri	Negative	
Anti-Yo	Negative	
RT-QUIC	Negative	
Neurological investigations		
CSF analysis		
Biochemistry		
Protein, mg/L	1600	150–500
Glucose, mmol/L	2.2	2.2–3.9
Cytology		
WCC, ×10^6^/L	43	
RBC, ×10^6^/L	21	
Mononuclear cell	100%	
Infectious screen	Negative culture/serology/viral PCR	
CSF flow cytometry	94% lymphoid origin, 96% CD5+ and T-cell predominant, too few B-cells	
EEG	Generalised slowing, no specific finding	
MR brain	Limited findings due to motion artefact, no discrete mass found	

Initial anterior pituitary hormone panel demonstrated a low thyroid stimulating hormone (TSH) and T4, as well as a mildly low testosterone level, with inappropriately normal luteinizing hormone (LH) and follicle stimulating hormone (FSH). In the absence of a pituitary lesion on MRI, the thyroid function abnormalities were thought to be due to non-thyroidal illness. His hyponatraemia was managed with 1.2 L fluid restriction, 1,200 mg salt tablets three times daily, and fludrocortisone 100 μg daily. He was commenced on a small dose of levothyroxine, 25 μg daily. A fluorodeoxyglucose-positron emission tomography (FDG-PET) scan did not demonstrate any FDG-avid lesions suggestive of haematological malignancy.

His condition continued to deteriorate throughout his hospitalisation despite correction of his serum sodium levels. On day 4 of his admission, he developed worsening confusion and dysautonomic symptoms, including hypotension, urinary retention, and constipation. Lumbar puncture for cerebrospinal fluid (CSF) analysis, infection screen and autoimmune encephalitis autoantibodies was performed, as well as an electroencephalogram (EEG). A short synacthen test (SST) demonstrated a normal adrenal response, with cortisol levels of 590 nmol/L at baseline, 749 nmol/L at 30 min and 901 nmol/L at 60 min.

Haematological malignancy remained a differential diagnosis for the patient’s presentation; however, the negative PET scan result made a diagnosis such as lymphoma less likely. Furthermore, the CSF analysis revealed normal cytology. Bone marrow aspiration and trephine biopsy was not performed, as a normal reticulocyte index suggested a normal marrow response, and his normocytic anaemia was thought to be due to chronic illness.

## Treatment

His hospital admission was further complicated by aspiration pneumonia and atrial fibrillation on day 12, resulting in type 1 respiratory failure requiring intubation. He was treated with intravenous antibiotics and intravenous methylprednisolone 1 gram for possible encephalitis. A repeat anterior pituitary hormone panel was ordered, which again demonstrated hypogonadotropic hypogonadism (testosterone: 0.5 nmol/L, FSH: 2.8 unit/L, LH: 0.8 unit/L) and now presumed secondary hypothyroidism (TSH: 0.23 mU/L, FT4: 5.8 pmol/L), although the hypogonadism was difficult to interpret in the setting of high-dose corticosteroid use.

Unfortunately, despite initial improvement with antibiotics and steroids, his condition was worsened by persistent tachyarrhythmia, bilateral pleural effusion, and hypotension refractory to inotropes. He passed away on day 24 of his hospital admission without a unifying diagnosis.

## Outcome and follow-up

**Figure 1:**
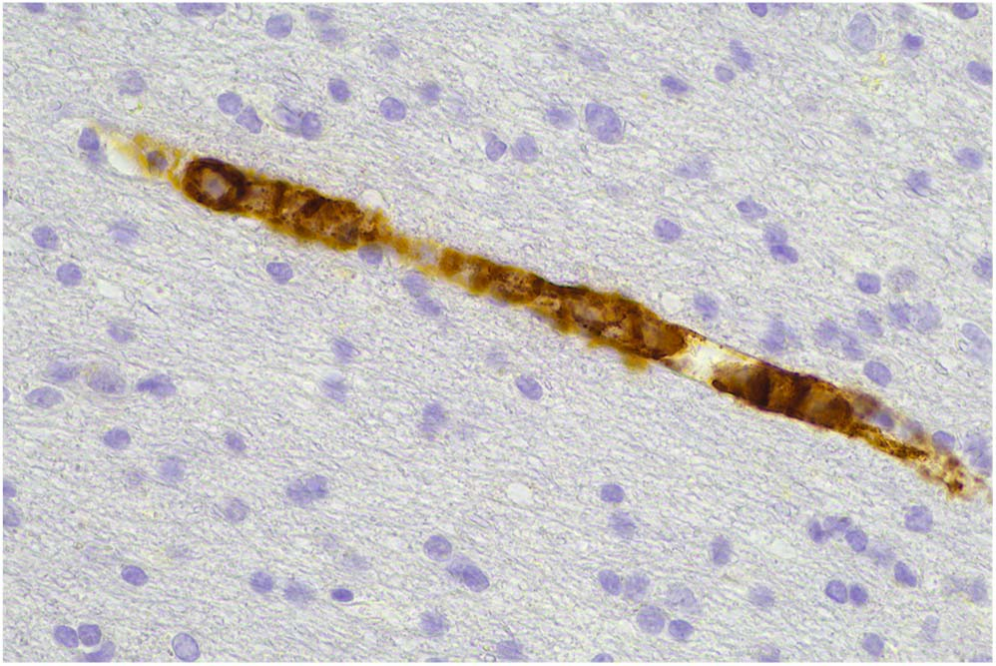
CD20 stain demonstrating intravascular B-cell lymphoma in dural vessel lumen.

Following the patient’s death, a post-mortem examination was conducted. The histological findings confirmed a diagnosis of intravascular lymphoma, with multi-organ involvement including brain, dura, pituitary, thyroid, and periadrenal soft tissues, among others (Fig. 1). There was also extravascular involvement of the bone marrow and spleen. Immunohistochemistry confirmed the lymphoma was of B-cell origin. Autopsy also confirmed hepatic and pulmonary congestion and oedema, as well as a likely lower urinary tract infection.

## Discussion

The unifying diagnosis in this case for our patient’s multiple endocrinopathies was intravascular lymphoma, involving multiple organs including the brain, dura and pituitary. Intravascular lymphoma is a rare diagnosis and clinically aggressive disease, with growth of lymphoma cells almost exclusively within small blood vessels (3). This growth can lead to the occlusion of blood vessels and failure of the supplied organs; however, the presenting clinical features are often vague and non-specific. As a result, it can be a challenging diagnosis to make, with historically a large majority of cases being diagnosed at autopsy, as with our case. However, rates of diagnosis while intervention is still possible are increasing, and treatment with immunochemotherapy, particularly rituximab, has been shown to improve outcomes (3).

In this particular case, our patient’s main presenting symptoms were vague and subacute in nature, including cognitive and functional decline, weight loss and headache. Other constitutional symptoms, including fever and night sweats, were not a prominent feature. Although his mild anaemia, thrombocytopaenia and elevated LDH, as well as the lytic lesions on CT, were suggestive of a haematological malignancy, the diagnosis remained elusive. Haematology opinion was sought; however, the negative FDG-PET scan result and absence of B-cells on cerebrospinal fluid cytology was felt to make the diagnosis of lymphoma unlikely. A bone marrow aspirate and trephine was not conducted for this patient, but may have had some diagnostic yield, given that extravascular involvement of the bone marrow was reported on the post-mortem examination.

There have been several case reports of intravascular lymphoma causing hypopituitarism, involving several or all pituitary axes (1, 4). This is thought to be due to vascular occlusion within the pituitary, resulting in reduction or loss of function (1). In our patient, his anterior pituitary function was partially affected, with central hypothyroidism and hypogonadotropic hypogonadism. It should be noted that while the patient had mild hypogonadotropic hypogonadism before hospital admission, more profound testosterone deficiency was only demonstrated after high-dose glucocorticoids were given, which may have confounded the results. Anila *et al.* (5) describe a case of panhypopituitarism in the setting of a pituitary mass, which was confirmed on histology to be intravascular lymphoma. In our case, however, there was no pituitary mass noted on MRI brain, and the pituitary was not reported to be enlarged at the time of autopsy.

The other major endocrinopathy in this case is hyponatraemia, which was thought to be secondary to the syndrome of inappropriate antidiuresis. SIAD can be associated with multiple pathologies, which require exclusion during the patient’s evaluation. A careful evaluation for causes such as malignancy, central nervous system pathology (including tumours, infection and trauma), lung pathology, and medications must be undertaken (6). In this case, malignancy was suspected as the underlying aetiology, given the clinical picture of weight loss, cytopaenias and spinal lytic lesions on CT imaging. SIAD has been described in association with intravascular lymphoma in several case reports (2, 4, 7, 8). The exact mechanism is unclear, but may result from the inappropriate release of antidiuretic hormone (ADH) by lymphoma cells, or the infiltration of lymphoma cells into the pituitary (4, 9). Authors such as Watabe *et al.* (9) also suggest that some inflammatory cytokines may stimulate ADH secretion and release by the hypothalamus.

This case presented a diagnostic challenge in terms of establishing a unifying diagnosis for multiple endocrinopathies, which originally presented with a constellation of vague and non-specific symptoms. The underlying diagnosis proved to be that of intravascular lymphoma affecting the pituitary, a rare condition that often goes unrecognised. While many of the patient’s presenting symptoms and biochemical abnormalities were noted to be endocrine issues, his underlying diagnosis was found to be a rare haematological condition, highlighting the importance of maintaining a broad and multisystem diagnostic approach for any undifferentiated presentation.

## Declaration of interest

The authors declare that there is no conflict of interest that could be perceived as prejudicing the impartiality of the research reported.

## Funding

This research did not receive any specific grant from any funding agency in the public, commercial, or not-for-profit sector.

## Patient consent

As the patient is deceased, written informed consent for publication of his clinical details was obtained from the patient’s wife.

## Author contribution statement

ZG and RD drafted the case report. AS and NN were treating physicians for the patient and were involved in conceptualisation and oversight of the case report, including editing and revision.
